# Changes Among Mexican Adults in Physical Activity and Screen Time During the COVID-19 Lockdown Period and Association With Symptoms of Depression, Anxiety, and Stress, May 29–July 31, 2020

**DOI:** 10.5888/pcd19.210324

**Published:** 2022-03-24

**Authors:** Alejandra Jáuregui, Gabriela Argumedo, César Hernández-Alcaraz, Alejandra Contreras-Manzano, Aaron Salinas-Rodríguez, Deborah Salvo

**Affiliations:** 1 Department of Physical Activity and Healthy Lifestyles, Center for Nutrition and Health, Cuernavaca, Morelos, Mexico; 2 Center for Evaluation Research and Surveys, National Institute of Public Health, Cuernavaca, Morelos, Mexico; 3 Prevention Research Center, Brown School, Washington University in St. Louis, St. Louis, Missouri

## Abstract

**Introduction:**

We examined the association between changes in physical activity and leisure screen time and mental health outcomes during the early stages of the recommended COVID-19 stay-at-home period in a national sample of Mexican adults aged 18 years or older.

**Methods:**

A cross-sectional online survey conducted from May 29 through July 31, 2020, among 1,148 participants, reported time spent in physical activity and leisure screen time during a typical week before (retrospectively) and a week during the COVID-19 stay-at-home period. Mental health outcomes during this period were measured with the Depression, Anxiety and Stress Scale (DASS-21). Linear regression models were used to estimate the associations between changes in physical activity and leisure screen time and mental health outcomes by socioeconomic status (SES), adjusting for potential confounders.

**Results:**

Compared with maintaining high levels of physical activity or increasing them, decreasing physical activity was associated with higher stress scores overall, and among people of high SES, with higher scores for DASS-21, depression, and anxiety. Among participants of low and medium SES only, increasing screen time was associated with higher DASS-21, depression, anxiety, and stress scores compared with maintaining low or decreasing leisure screen time.

**Conclusion:**

Results highlight the potential protective effect of physical activity and limited leisure screen time on mental health in the context of COVID-19 stay-at-home restrictions.

**Summary**

**What is already known on this topic?**

Changes in physical activity and leisure screen time during the COVID-19 pandemic have been related to mental disorders in high-income countries. Differences by socioeconomic status have not been reported for Latin American countries.

**What is added by this report?**

During the recommended stay-at-home period, associations between changes in physical activity and leisure screen time with mental health outcomes were modified by socioeconomic status.

**What are the implications for public health practice?**

Interventions to reduce sedentary behaviors and promote physical activity to improve adults’ mental well-being during the COVID-19 stay-at-home period should be prioritized among those living in disadvantaged socioeconomic conditions.

## Introduction

Engaging in regular physical activity and limiting sedentary time can improve mental health (1). However, the COVID-19 pandemic has not only changed the way in which people are physically active, but it has decreased the length of time spent being active. In high-income countries, these changes have led to an increase in symptoms of depression, anxiety, and stress (2,3). Increased screen time has also negatively affected mental health (3,4). Although information on these changes is available for high-income countries outside Latin America, little is known about how lifestyle changes resulting from the pandemic have affected populations in Latin America. Studies are needed that examine the impact of stay-at-home orders on physical activity, sedentary time, and other health outcomes, as well as on socioeconomic inequalities (5). In Mexico, pandemic mitigation measures began in March 2020 with initial stay-at-home recommendations and workplace closures for all nonessential activities. By April 2021, Mexico ranked among the leading countries in the world for COVID-19 deaths per capita (6).

The pandemic has had profound effects on the physical inactivity epidemic (7). Stay-at-home restrictions have reduced opportunities for physical activity, and social isolation has led people to spend large periods of time indoors and in small quarters, with an increase in leisure screen time (eg, using mobile devices, watching television). Both physical inactivity and high levels of screen time are known to precede negative effects on mental health (8).

Physical activity in Latin America is mainly driven by necessity (utilitarian physical activity) and by the need for social interaction (leisure-based activity), more than by health or fitness benefits (9,10). In Latin America, including Mexico, most urban residents cannot afford a motor vehicle, making active transportation the only option for most people (10). Active travel, especially walking and bicycling, may have positive emotional effects, in part because of social interactions (11,12). Studies suggest that among Mexican adults, travel-related physical activity is an important contributor to overall physical activity, mainly among low-income people. Meanwhile, health-motivated physical activity (ie, physical activity performed at a gymnasium) is more prevalent among high-income people (13). Furthermore, Latin Americans tend to choose leisure-time physical activity that is social (9), and use informal settings not oriented to exercise or sports (eg, shopping malls) for physical activity (13,14), probably motivated by the need to interact with others. Hence, decreases in physical activity caused by stay-at-home recommendations could be affecting the mental health of Mexican adults, both because of physiologic changes (ie, exercise releases endorphins, which in turn trigger positive feelings) and in response to sociocultural factors. Furthermore, residents of affluent neighborhoods are likely to have enough space at home to exercise, whereas those living in low-income neighborhoods may rely more on public spaces to be active. Hence, the limitations COVID-19 has imposed on the use of public places may affect low-income residents to a greater extent.

Our study’s objective was to evaluate the association between changes in levels of physical activity and leisure screen time and symptoms of depression, anxiety, and stress among adults in Mexico during the early stages of the COVID-19 stay-at-home recommendation. We also explored how these associations differed by socioeconomic status (SES).

## Methods

### Study design

We conducted an online survey to assess self-reported physical activity, leisure screen time, and mental health before and during the COVID-19 stay-at-home period in Mexico among a non-probabilistic sample of adult (≥18 y) internet users. In Mexico, 72% of the Mexican population has access to the internet, most users are aged 18 to 40 years, and most are in the middle and high socioeconomic levels (15). Data were collected from May 29 (2 months after the beginning of the COVID-19 stay-at-home recommendations in Mexico) through July 31, 2020, when the first wave of COVID-19 was increasing but had not yet plateaued. The study was reviewed and approved by the Research and Ethics Commissions at the Mexican National Institute of Public Health (approval no. 1661).

### Participants and selection process

Participants were recruited through the web pages and social media sites of 2 organizations, the National Institute of Public Health and the Latin American Congress for Physical Activity and Health Research. Two Facebook advertisements were placed to increase the number of people reached across all regions in Mexico. Participants provided informed consent online before completing the survey. 

### Changes in physical activity

We used a questionnaire to assess self-reported physical activity among people we hypothesized to be affected by stay-at-home restrictions before and during the pandemic. The questionnaire was based on selected items from the self-administered past-week Modifiable Activity Questionnaire (SMAQ) (16) and was culturally and linguistically adapted for Mexico. Participants self-reported the frequency and duration of the following leisure-time activities: walking, bicycling, running, online physical activity classes, aerobic exercises (treadmill, stationary bicycle, stair climber) at home or at a gym, muscle-strengthening activities (eg, weightlifting) at home or at a gym, sports (eg, tennis, basketball, soccer), active videogames (eg, Wii Dance games), swimming or other water activities, and transport-related physical activities (ie, walking and bicycling), before and during the time that a typical week of the stay-at-home recommendation was in effect. We derived activity-specific and overall leisure-time minutes per week. Participants were classified as meeting the physical activity recommendations of the World Health Organization (17) if they reported engaging in at least 150 minutes per week of moderate to vigorous physical activity, both before and during the stay-at-home recommendation period. Next, participants were classified into groups according to changes: decreasing physical activity (meeting recommendations before but not during the stay-at-home period), maintaining inactive status (not meeting recommendations before nor during the stay-at-home period), and maintaining or increasing physical activity (meeting recommendations during the stay-at-home period). This last category considered participants who maintained or increased their physical activity, based on the low percentage in the increasing physical activity group.

### Changes in leisure screen time

A similar survey approach was used to measure leisure screen time. Participants self-reported the frequency and duration of leisure screen time during a typical day, before and during the recommended stay-at-home period. On the basis of previous studies (17), participants were classified as engaging in less than 2 hours or more than 2 hours per day of leisure screen time, both at baseline and during the stay-at-home period. Participants were then classified into groups according to changes in screen time: increased screen time (<2 h/d before and ≥2 h/d during the stay-at-home period), maintained high screen time (≥2 h/d before and during the stay-at-home period), or maintained low or decreasing screen time (<2 h/d during the stay-at-home period).

### Depression, anxiety, and stress symptoms

We used the Spanish version of the 21-item Depression, Anxiety and Stress Scale (DASS-21) (18), a set of 3 self-report subscales designed to measure symptoms of depression, anxiety, and stress over the past week. Each 7-item subscale is rated on a 4-point Likert scale ranging from 0 (Did not apply to me at all) to 3 (Applied to me very much or most of the time). Subscale scores are calculated by adding the numbers associated with response options for 7 subscale items (range 0–21). The total score was calculated by adding the totals of the 3 subscale scores, with higher scores representing greater symptoms of depression, anxiety, or stress. We also identified the presence of severe and very severe symptoms of depression, anxiety, or stress by using the cutoff points recommended for DASS-21 (19). DASS-21 has shown adequate psychometric properties of validity, internal consistency, test–retest reliability, and construct validity in various countries and cultures (20) and has been used to assess mental health around the globe during the COVID-19 pandemic (21). 

### Sociodemographic characteristics

We collected information on age, sex, education completed, and marital status. Household SES was measured with a validated questionnaire, and participants were categorized as either high SES or middle or low SES (22). Other household characteristics were the presence of children aged 6 years or younger and the square meters of living space per inhabitant (<22 m^2^, 22–38 m^2^, ≥38 m^2^). We also explored the amount of time per day in home confinement (≤19 hr/d or >19 hr/d) and changes in the participant’s work during the pandemic.

### Data analysis

The study sample size was powered to examine differences in the proportion of adults meeting physical activity recommendations or spending less than 2 hours per day in leisure screen time. We estimated that with a sample size of 528 participants, our study had an estimated 80% power to detect a 5% difference in this proportion.

Means and SDs and proportions were used to describe time spent in physical activity and leisure screen time before and during the stay-at-home period. The difference between these 2 times was calculated to estimate changes in physical activity and leisure screen time from before to during the stay-at-home period.

We performed linear regression models to analyze the associations between physical activity changes and mental health outcomes during the pandemic. Separate models were used for the DASS-21 score and its 3 subscales. Robust SE estimates were used because score distributions of DASS-21 and its subscales were right-skewed. All models were adjusted for sociodemographic characteristics and changes in leisure screen time or physical activity (ie, models exploring the association between physical activity changes were adjusted for leisure screen time, and vice versa). Additionally, because exploratory analyses indicated differences by SES in the association between outcomes and exposures, overall sample as well as stratified models were analyzed by SES. All data preparation and analyses were conducted with Stata 15.0 (StataCorp LLC). Differences were considered significant at *P* < .05.

## Results

In total, 2,096 adults accessed the questionnaire link and consented to participate, and 1,619 (77.0%) completed the survey. After eliminating participants with missing information for any of the analytical variables (n = 471), a total of 1,148 participants were included in the final analysis. Most were women (77.6%) with no children aged 6 years or younger living at home (87.3%). Most were confined to home (78.9%). About half were classified as low or middle SES (55.8%), single (51.3%), with an undergraduate degree (42.2%), and with no changes in their job during the COVID-19 pandemic (52.0%). A total of 35.3% were aged 18 to 29 (Table 1). During the stay-at-home period, from 10% to 12% had severe or very severe symptoms of depression, anxiety, or stress (Table 1). Differences across SES were observed for sex, age group, education level, having children aged 6 years or younger, marital status, living area per person in the household, and the average of the depression score (*P* < .05). 

**Table 1:** Demographic and Mental Health Characteristics of Mexican Adults (N = 1,148) Before and During COVID-19 Stay-At-Home Period, by Socioeconomic Status, Mexico, May 29–July 31, 2020

Characteristic^a^	Total sample (N = 1,148)	55.8%	44.2%	*P* value^b^
		Low or medium SES (n = 641)	High SES (n = 507)	
**Demographics**				
Sex				
Female	77.6	77.1	78.3	.048
Male	22.4	22.9	21.7	
Age, y				
18–29	35.3	40.2	28.9	<.001
30–39	33.7	32.7	35.1	
40–49	19.1	17.9	20.7	
50–59	8.9	6.3	12.2	
≥60	2.8	2.6	2.9	
Education completed level				
High school diploma or less	25.7	40.2	7.3	<.001
Undergraduate degree	42.2	45.5	38.1	
Postgraduate degree	32.1	14.2	54.6	
Children in household aged <6 y				
No	87.3	85.6	89.5	.048
Yes	12.6	14.4	10.4	
Job status				
No changes	52.0	50.7	53.6	.20
I kept my job, but continued working from home	21.8	21.3	22.5	
I kept my job, but my salary was reduced	8.1	7.4	8.8	
I kept my job, but without pay, or had to close my business	7.5	8.4	6.5	
I lost my job, or other	10.4	12.0	8.4	
Marital status				
Single	51.3	55.1	46.7	.005
Married/cohabiting	41.3	37.1	46.7	
Divorced or widowed	7.2	7.8	6.5	
Area per person in household				
<22 m^2^	37.6	48.3	24.1	<.001
22 to <38 m^2^	29.8	27.9	32.3	
≥38 m^2^	32.4	23.7	43.6	
Home confinement				
Yes	78.9	78.7	79.1	.87
No	21.1	21.3	20.9	
**Physical activity**				
Maintain active or increase physical activity	71.3	68.5	74.9	.26
Maintain inactive	8.7	9.3	7.8	
Decrease physical activity	19.9	22.1	17.2	
**Leisure screen time**				
Maintain low or decrease screen time	12.6	12.3	13.0	.61
Maintain high screen time	66.4	66.3	66.6	
Increase screen time	20.9	21.3	20.3	
**Mental health**				
Depression, Anxiety and Stress Scale^c^				
Score, mean (SD)	13.2 (11.9)	13.8 (12.1)	12.4 (11.6)	.06
Depression^c^				
Score, mean (SD)	8.2 (9.0)	8.7 (9.3)	7.6 (8.6)	.05
Yes	10.6	11.5	9.4	.26
Anxiety^c^				
Score, mean (SD)	6.2 (7.7)	6.4 (7.8)	5.7 (7.5)	.12
Yes	12.0	12.8	11	.85
Stress^c^				
Score, mean (SD)	12.0 (9.4)	12.4 (9.5)	11.4 (9.3)	.08
Yes	10.4	10.7	9.8	.62

Abbreviation: SES, socioeconomic status.

a Values are percentage unless otherwise indicated.

b *P* values calculated by using χ^2^ tests; values are significant at *P* < .05.

c Depression, Anxiety and Stress Scale (18), scores range from 0 (no symptoms) to 63 (greater symptoms); subscales range from 0 (no symptoms) to 21 (greater symptoms).

### Changes in physical activity and leisure screen time

During the early stages of the stay-at-home period, total physical activity decreased about 23%, whereas total leisure screen time increased about 70% (*P* < .01 for both) (Table 2). Before the start of the stay-at-home period, 8 in 10 participants met physical activity recommendations, and 3 in 10 had <2 hours per day of leisure screen time. During the stay-at-home period, 7 in 10 participants met physical activity guidelines and 1 in 10 spent less than 2 hours per day in leisure screen time. Decreases in most activity types were reported, with the largest ones for walking for leisure or for transport and aerobic or strength activities at a gymnasium. In contrast, increases in online physical activities, playing active videogames, and aerobic and strength activities at home were observed.

**Table 2:** Physical Activity and Leisure Screen Time Among Mexican Adults (N = 1,148) Before and During COVID-19 Stay-At-Home Period, Mexico, May 29–July 31, 2020

Variable^a^	Before stay-at-home period	During stay-at-home period	Difference	*P* value^b^
Physical activity				
Total physical activity (min/wk)	646.7 (621.8)	497.1 (587.1)	−149.6 (549.8)	.001
Walking for leisure	157.3 (201.9)	39.0 (123.5)	−118.3 (209.4)	
Walking for transport	101.8 (174.3)	15.2 (46.6)	−86.6 (168.2)	
Bicycling for leisure	23.0 (87.3)	7.9 (56.6)	−15.1 (80.5)	
Bicycling for transport	9.3 (47.8)	5.1 (35.1)	−4.2 (49.1)	
Online physical activity	32.6 (98.0)	172.9 (263.5)	140.3 (258.2)	
Running	55.7 (115.7)	16.6 (74.8)	−39.1 (122.4)	
Aerobic activities at gym	94.5 (183.9)	23.1 (88.3)	−71.4 (188.8)	
Aerobic activities at home	22.9 (68.6)	65.8 (130.0)	42.9 (121.0)	
Strength activities at gym	93.7 (187.8)	26.9 (114.5)	−66.8 (174.0)	
Strength activities at home	17.5 (59.8)	72.4 (134.2)	54.9 (126.2)	
Sports time	24.0 (100.2)	8.0 (91.9)	−16.0 (96.3)	
Playing active video games	14.4 (91.6)	44.0 (186.6)	29.6 (166.5)	
Swimming	21.2 (66.6)	2.6 (24.4)	−18.6 (66.0)	
Meeting WHO physical activity guidelines, % (95% CI)	83.6 (81.3–85.6)	71.3 (68.6–73.9)	−12.3 (51.1)	
Leisure screen time				
Total leisure screen time (h/d)	3.1 (2.2)	5.0 (3.0)	1.9 (2.2)	.001
Less than 2 h/d of leisure screen time, % (95% CI)	31.5 (28.9–34.3)	12.6 (10.8–14.6)	−18.9 (43.9)	

Abbreviation: WHO, World Health Organization.

a Values are mean (SD) unless otherwise indicated.

b *P* values calculated by using bivariate regression models; values are significant at *P* < .05.

### Changes in physical activity and mental health during the stay-at-home period

We found evidence of effect modification by SES in relation to physical activity and mental health (Table 3). Among participants of low and medium SES, those in the decreasing physical activity group had higher stress scores (β = 2.36; 95% CI, 0.68–4.04) compared with those in the group that maintained active or increased physical activity. Compared with those in the group that maintained active or increasing physical activity, respondents in the group that maintained inactive physical activity status had higher scores for most mental health outcomes except for anxiety: (DASS-21: β = 4.41, 95% CI, 0.94–7.88; depression: β = 3.48, 95% CI, 0.60–6.35; stress: β = 3.36, 95% CI, 0.66–6.06). 

**Table 3:** Adjusted^a^ and Stratified^b^ Associations Between Mental Health Outcomes, Socioeconomic Status, and Changes in Physical Activity or Leisure Screen Time Among Mexican Adults (N = 1,148) During the COVID-19 Stay-at-Home Period, Mexico, May 29–July 31, 2020

Change category	Total sample, N = 1,148		Low or medium SES b(n = 641)		High SES^b^ (n = 507)	
	β (95% CI)	*P* value^c^	β (95% CI)	*P* value^c^	β (95% CI)	*P* value^c^
**Physical activity**						
Depression, Anxiety and Stress Scale^d^						
Maintain active or increase physical activity	1 [Reference]		1 [Reference]		1 [Reference]	
Maintain inactive	3.70 (1.16 to 6.24)	.004	4.41 (0.94 to 7.88)	.013	2.67(−1.18 to 6.52)	.17
Decreased physical activity	3.77 (1.98 to 5.56)	<.001	2.08 (−0.20 to 4.17)	.052	6.74 (3.57 to 9.90)	<.001
Depression^d^						
Maintain active or increase physical activity	1 [Reference]		1 [Reference]		1 [Reference]	
Maintain inactive	2.79 (0.73 to 4.84)	.008	3.48 (0.60 to 6.35)	.018	1.55 (−1.46 to 4.56)	.31
Decrease physical activity	1.98 (0.69 to 3.27)	.003	0.73 (−0.83 to 2.29)	.358	4.20 (1.93 to 6.46)	<.001
Anxiety^d^						
Maintain active or increase physical activity	1 [Reference]		1 [Reference]		1 [Reference]	
Maintain inactive	1.55 (−0.76 to 3.18)	.062	1.98 (−0.19 to 4.15)	.074	1.04 (−1.46 to 3.53)	.42
Decrease physical activity	2.39 (1.17 to 3.62)	<.001	1.06 (−0.38 to 2.50)	.150	4.66 (2.54 to 6.77)	<.001
Stress^d^						
Maintain active or increase physical activity	1 [Reference]		1 [Reference]		1 [Reference]	
Maintain inactive	3.06 (1.07 to 5.06)	.003	3.36 (0.66 to 6.06)	.015	2.76 (−0.30 to 5.81)	.08
Decrease physical activity	3.18 (1.78 to 4.58)	<.001	2.36 (0.68 to 4.04)	.006	4.62 (2.19 to 7.05)	<.001
**Leisure screen time**						
Depression, Anxiety and Stress Scale^d^						
Maintain low or decrease screen time	1 [Reference]		1 [Reference]		1 [Reference]	
Maintain high screen time	3.88 (2.34 to 5.42)	<.001	4.69 (2.74 to 6.63)	<.001	2.42 (−0.02 to 4.87)	.05
Increase screen time	3.74 (1.82 to 5.67)		6.82 (4.13 to 9.52)		−0.68 (−3.37 to 2.01)	.62
Depression^d^						
Maintain low or decrease screen time	1 [Reference]		1 [Reference]		1 [Reference]	
Maintain high screen time	2.68 (1.58 to 3.79)	<.001	2.98 (1.63 to 4.33)	.001	1.94 (0.17 to 3.72)	.03
Increase screen time	2.80 (1.40 to 4.21)	<.001	4.92 (2.95 to 6.89)	<.001	−0.20 (−2.12 to 1.73)	.84
Anxiety^d^						
Maintain low or decrease screen time	1 [Reference]		1 [Reference]		1 [Reference]	
Maintain high screen time	2.41 (1.36 to 3.45)	<.001	2.82 (1.39 to 4.26)	<.001	1.61 (−0.02 to 3.23)	.05
Increase screen time	2.21 (0.89 to 3.53)	.001	3.94 (2.00 to 5.88)	<.001	−0.37 (−2.17 to 1.43)	.68
Stress^d^						
Maintain low or decrease screen time	1 [Reference]		1 [Reference]		1 [Reference]	
Maintain high screen time	2.67 (1.34 to 4.01)	<.001	3.57 (1.88 to 5.26)	<.001	1.30 (−0.86 to 3.46)	.24
Increase screen time	2.47 (0.86 to 4.08)	.003	4.80 (2.61 to 6.98)	<.001	−0.79 (−3.16 to 1.58)	.51

Abbreviation: SES, socioeconomic status.

a Betas and 95% Cis estimated with linear regression models, adjusted for demographic variables (sex, age, education, children <6 years in household, job status, marital status, and area per person in household), and changes in physical activity or leisure screen time, accordingly.

b Models stratified by SES. Preliminary analysis showed differences by SES.

c *P* values calculated by using linear regression models; values are significant at *P* < .05.

d Depression, Anxiety and Stress Scale scores range from 0 (no symptoms) to 63 (greater symptoms); subscales range from 0 (no symptoms) to 21 (greater symptoms).

Among participants of high SES, those in the group with decreasing physical activity had higher scores (ie, worse mental health) for all mental health outcomes: (DASS-21: β = 6.74, 95% CI, 3.57–9.90; depression: β = 4.20, 95% CI, 1.93–6.46; anxiety: β = 4.66, 95% CI, 2.54–6.77; stress: β = 4.62, 95% CI, 2.19–7.05) compared with those from the group that maintained active or increasing physical activity.

### Changes in leisure screen time and mental health during the COVID-19 pandemic

We found evidence of effect modification by SES in relation to screen time and mental health (Table 3). Among low or medium SES participants, increasing or maintaining high levels of screen time during the pandemic was significantly associated with higher DASS-21 scores. For the group increasing screen time, values were β = 6.82; 95% CI, 4.13–9.52; values for the group maintaining high levels of screen time were β = 4.69; 95% CI, 2.74–6.63. For depressive symptoms, values for the group increasing screen time were β = 4.92; 95% CI, 2.95–6.89; for the group maintaining high levels of screen time, values were β = 2.98; 95% CI, 1.63–4.33. For anxiety, values for the group increasing screen time were β = 3.94; 95% CI: 2.00–5.88; for those maintaining high levels of screen time, values were β = 2.82; 95% CI: 1.39–4.26. For stress, values for the group increasing screen time were β = 4.80; 95% CI, 2.61–6.98; for the group maintaining high levels of screen time, values were β = 3.57; 95% CI, 1.88–5.26. All associations, with the exception of the group that maintained high levels of screen time (β = 1.94; 95% CI, 0.17–3.72), were nonsignificant for respondents of high SES.

## Discussion

Our findings support the inverse association between physical activity and symptoms of anxiety, stress, and depression during the early stages of the COVID-19 pandemic. Conversely, results indicate an association between increased leisure screen time and worse mental health outcomes. These associations were modified by SES level.

Physical activity is a well-known behavior for supporting mental health (1). We found that participants who maintained or increased their physical activity during the stay-at-home period had lower levels of anxiety, stress, and depression than those who did not, echoing studies suggesting that physical activity is a relevant behavior when addressing mental health outcomes in the context of the pandemic (23). Results of our study also suggest that, among Mexican adults, the mechanisms by which changes in physical activity during confinement to home may be associated with mental health outcomes vary by SES. For instance, decreases in physical activity levels during the pandemic appear to be associated with mental health among Mexicans of both high and low or medium SES. Among those of low or medium SES, the association between decreasing levels of physical activity and mental health was mainly due to high levels of stress, with no observable associations with depression or anxiety symptoms. Physical activity has been linked to a reduced psychological responsiveness to physical and psychosocial stressors, resulting in psychological and physiological benefits that enable people to cope with stress more effectively (24). Secular changes in social interaction may also help explain this association. Studies in Mexico suggest that having low SES is associated with high levels of travel-related physical activity and the use of public spaces (ie, streets and parks) and places that are not exercise- or sports-based for physical activity (eg, shopping malls) (13). These activities and spaces are known to provide opportunities for social interaction (14). Decreases in physical activity among low-income groups may also substantially reduce social interaction. Limited access to public places for physical activity may also account for these associations. A recent qualitative study among Mexico City residents reported that although the use of urban green spaces decreased among all participants during the COVID-19 pandemic, those in low-income groups were more affected because they avoided using public transportation, their main transportation mode, to get to these places (25). As proposed by others, the social context of physical activity may be important in reducing the risk of poor mental health (13,14,25).

In contrast, and opposite to our initial hypothesis, decreases in physical activity levels were consistently and strongly related to higher anxiety, stress, and depression scores among high-SES participants compared with low- and middle-SES participants. Given the more health-driven nature of participation in physical activity among people of high SES (13), those people possibly may be more susceptible to declines in mental health outcomes in response to sudden decreases in their physical activity levels. Also, increased anxiety, depression, and stress possibly contributed to reduced physical activity. Overall, findings indicate that the COVID-19 pandemic may have limited the use of physical activity as a mental health coping mechanism for many. This underscores the need to ensure accessibility to different physical activity opportunities for all, even in times of crisis. Government responses to the COVID-19 pandemic play a major role in providing these opportunities. Optimizing public spaces for physical activity (eg, new pedestrian and cycling infrastructure) may be useful for increasing physical activity and reducing mental health inequalities during the pandemic. Internet-based cognitive behavior therapy and physical activity may also be effective treatment alternatives for those with mildly to moderately poor mental health (26). It must be noted that the lack of control that people have over lockdown orders, which restricted their access to public open spaces, and how these lockdown orders had differential effects across people of different SES levels, can in itself be an important determinant of mental and physical health. Future studies should explore the role that lack of control over major, top-down policy and environmental changes may have had on physical and mental health during the pandemic.

Conversely, results of our study showed that increased leisure screen time was associated with increased symptoms of stress, anxiety, and depression during the stay-at-home period, as suggested by a review study identifying excessive sedentary time as a risk factor for depression in adults (27). Findings from our study also indicate that this association differs across socioeconomic levels, with consistent associations between increased screen time during the stay-at-home period and higher depression, anxiety, and stress scores among low- and medium-SES participants, but not among their peers with high SES. In the context of the COVID-19 pandemic, increases in leisure screen time may be due to various reasons, including the need for more information about lockdown procedures and disease treatment or outcomes, the need to connect with others, or internet addiction (28). Also, participants of low SES possibly engaged in more mentally passive sedentary behaviors (ie, with no mental activity requirements), like watching television, than high-SES participants did, as suggested by a previous study (29). Mentally passive sedentary behaviors have been associated with a higher risk of depression, whereas mentally active sedentary behaviors, such as reading, may protect against the onset of depression (30). Although we asked participants to report their total leisure screen time, we were unable to capture the nature of their sedentary activities. Further studies investigating the effects of different types of sedentary behaviors on mental health are needed. In a broader sense, results of our study align with those suggesting that the pandemic has exacerbated social and health inequalities (7); in light of this, policy makers and decision makers at local, provincial, and national levels need to recognize the importance of physical activity and screen time for mental well-being and to introduce regulatory responses, even when mobility restrictions are involved. In our study leisure screen time increased across categories of physical activity changes, suggesting that increases in screen time are widespread and may be independent of active behaviors. Thus, strategies aiming to mitigate pandemic–related mental health effects should include strategies addressing both physical activity and screen time. In line with recent guidelines (17), interventions aiming to replace sedentary behaviors with physical activity of any intensity have shown promising results for promoting good mental health (31).

Our study provides original data from adults across Mexico and to our knowledge is the first study focused on the association between changes in physical activity and leisure screen time on mental health during the COVID-19 pandemic stay-at-home period across a large sample of Latin American adults. Changes in these behaviors threaten the physical and mental health of Mexican adults, especially because of the uncertainty of the length of social isolation required to prevent the spread of COVID-19. Knowledge gained from our study may inform interventions aimed at improving the mental well-being of adults in Mexico or other countries that share similar socio-contextual conditions.

Our study had several limitations. First, although participants were asked to retrospectively report their physical activity and screen time before and during the pandemic, the cross-sectional design of the survey precludes inferring causality. The reported associations are possibly partly explained by reverse causality. Furthermore, data were collected at the initial stages of the COVID-19 pandemic in Mexico. Future studies should address variations in the measured behaviors for longer periods of time or at different time points of the stay-at-home period, which might differ from findings reported here. The stay-at-home period itself limited the possibility of using device-based measures of physical activity, which provide more accurate estimates of both physical activity and sedentary behavior (32). We used a self-reported questionnaire designed to measure the underlying continuity of severity of psychiatric symptoms, which is not intended to establish a psychiatric diagnosis as would many clinical measures that may be considered diagnostic “gold standards” (eg, structured clinical interview and functional neuroimaging) (33). Similarly, our conclusions are based on self-reports, which are prone to overestimation, especially among people with severe mental illness (34). Additionally, measures included a non-exhaustive list of physical activity types, and although they are based on a previously validated instrument (SMAQ) (16), the new version has not been validated for use in Mexico. Characteristics of the study sample resemble those of internet users in Mexico (15). Compared with national estimates, participants had a similar prevalence of physical inactivity and depressive symptoms (35). However, because of the nature of the online survey and the fact that ours was a nonprobabilistic sample, participants had a higher socioeconomic status and education level than the general Mexican population (35). Thus, the representativeness of our study is limited to the analytic sample. Despite this, our study uncovered possible differential effects of movement behaviors on mental health among adults from different socioeconomic backgrounds that merit further investigation. Finally, our recruitment strategy may have precluded our including people with severe and very severe depression, anxiety, or stress.

Results of our study highlight the favorable effects of physical activity and limited leisure screen time on mental health well-being in the context of COVID-19 stay-at-home restrictions. Customized interventions to reduce sedentary behaviors and promote physical activity could improve the mental health of adults during similar stay-at-home restrictions. Concerted efforts should be prioritized among people living in disadvantaged socioeconomic conditions with limited access to leisure physical activity opportunities.
